# When Rare Meets Reality: A Case of Kikuchi-Fujimoto Disease in Tertiary Care Center

**DOI:** 10.7759/cureus.66747

**Published:** 2024-08-13

**Authors:** Indrika Sharma, Raju K Shinde, Venkatesh M Rewale, Tushar Nagtode, Akshunna Keerti

**Affiliations:** 1 General Surgery, Jawaharlal Nehru Medical College, Datta Meghe Institute of Higher Education and Research, Wardha, IND; 2 Internal Medicine, Jawaharlal Nehru Medical College, Datta Meghe Institute of Higher Education and Research, Wardha, IND

**Keywords:** rare disease, lymphadenopathy, kikuchi-fujimoto disease, histiocytic necrotizing lymphadenitis, autoimmune diseases

## Abstract

Kikuchi-Fujimoto disease is a very rare disease, basically involving young adults and ubiquitously distributed. It is characterized by fever and benign lymph node swelling. The distinguishing features of this disease are cervical lymphadenopathy, constitutional symptoms resembling tuberculosis, and its penchant to affect young people of Oriental or Asian descent, especially women. We describe an instance of a 42-year-old female who arrived with multiple neck swellings. On physical examination, there was palpable right-sided cervical lymphadenopathy, while laboratory investigations were essentially within normal limits except for raised erythrocyte sedimentation rate and anemia. After cefepime and nonsteroidal anti-inflammatory medications were administered, symptoms subsided, and lymphadenopathy receded in the patient. This case supports the importance of histological evaluation to reach an exact diagnosis and guide treatment and the need to consider Kikuchi-Fujimoto disease in the differential diagnosis of lymphadenopathy.

## Introduction

Histiocytic necrotizing lymphadenitis, also known as Kikuchi-Fujimoto disease (KFD), is a very rare idiopathic process [1]. In 1972, Japanese researchers Seishi Kikuchi and Y. Fujimoto reported the disease independently of each other and described it as a rare benign disorder. This was the first description regarding KFD [2]. Most of the patients have firm to rubbery cervical lymphadenopathy with intermittent pyrexia; more severely affected patients may present with leucopenia, splenomegaly, weight loss, and a high erythrocyte sedimentation rate (ESR) [3]. In some patients, non-specific skin lesions may be seen. Adequate correlation between clinical and histopathologic findings may allow for the early diagnosis of Kikuchi's disease and avoid unnecessary diagnostic investigations and inappropriate management of the same [4]. This is a self-limiting illness and is usually treated with nonsteroidal anti-inflammatory medicines, with corticosteroids and hydroxychloroquine used in more aggressive diseases [2]. Cervical necrotizing lymphadenitis was seen in this 42-year-old female patient at the period of the first report.

## Case presentation

A 42-year-old female reported to the surgery ward with a 45-day history of multiple swellings on the right side of her neck. The swellings were initially small (0.5 × 0.5 cm) and painless, with an insidious onset and gradual progression in size. She denied any associated pain or discharge from the swellings. The patient reported a history of contact with her sister, who had tested positive for *Mycobacterium tuberculosis*. Routine investigations on the day of admission were normal: hemoglobin was 10.3 gm/dL, white blood cells were 4,800/cumm, and platelets were 2.33 lacs/cumm. Hemogram and biochemical parameters from blood tests fell within normal ranges (Table 1), apart from a raised ESR of 86 mm/hour and anemia.

**Table 1:** Laboratory investigation on the day of admission

Investigation Name	Patient Value	Reference Value
Hemoglobin (Hb)	10.3 mg/dL	11-15 mg/dL
Red blood cell (RBC)	4.97 million/cumm	3.8-5.8 million/cumm
Hematocrit (HCT)	32.7%	36-44%
Erythrocyte sedimentation rate (ESR)	86 mm/hour	<20 mm/hour
Mean corpuscular volume (MCV)	72 fL	76-96 fL
Mean cell hemoglobin (MCH)	22 pg	27-32 pg
Mean corpuscular hemoglobin concentration (MCHC)	31.5 g/dL	31-35 g/dL
Platelet count	2.33 lacs/cumm	1.5-4 lacs/cumm
Red cell distribution width (RDW)	14.2%	1-15%
Total leukocyte count (TLC)	4,800/cumm	4,000-11,000/cumm
Differential leukocyte count		
Neutrophils	55%	40-75%
Lymphocytes	40%	20-45%
Eosinophils	2%	1-6%
Monocytes	3%	2-10%
Basophils	0%	0-1%
Coagulation profile		
Activated partial thromboplastin time (APTT)	36.7 seconds	29.5 seconds
Prothrombin time (PT)	13.4 seconds	11.9 seconds
International normalized ratio (INR)	1.13	0.8-1.2

**Figure 1:**
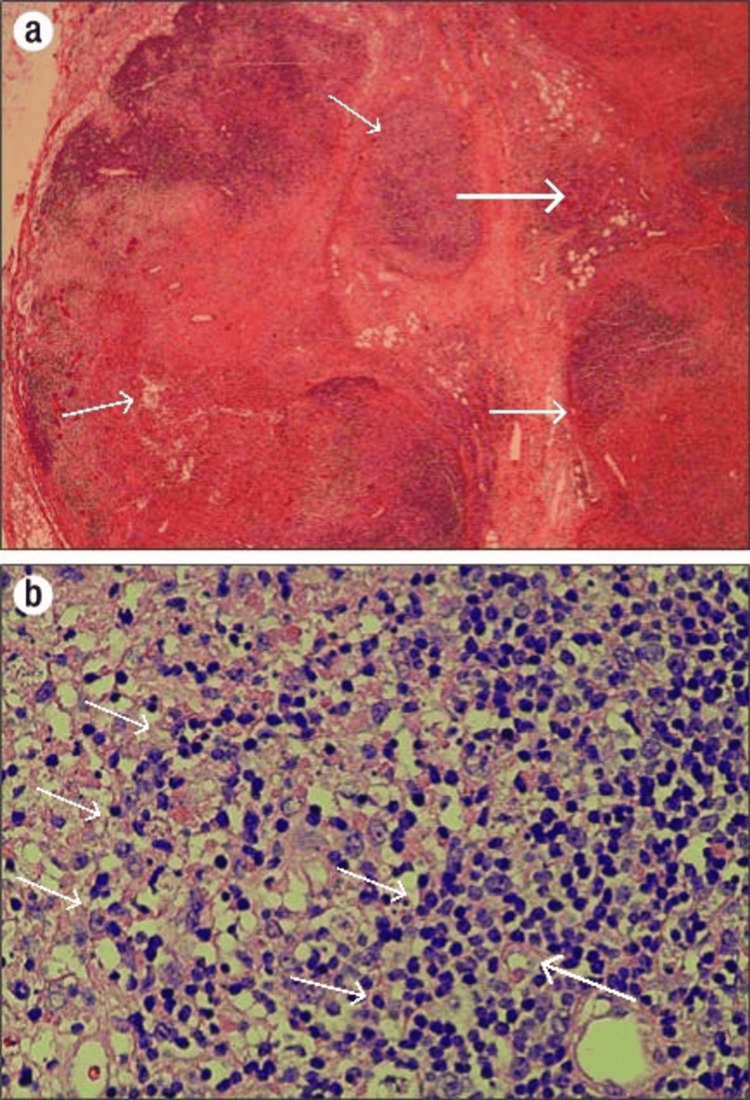
A) Complete effacement of nodal architecture with an area of necrosis surrounded by preserved lymphoid tissue shown by arrows. The necrosis is patchy and well-circumscribed and does not contain neutrophil (H&E low power). B) Irregular, pale areas composed of histiocytes, eosinophilic granular material, and karyorrhectic cell debris surrounding a necrotic zone shown by arrows. Lymphocytes, along with immunoblasts, are also seen. No neutrophils, eosinophils, or plasma cells are appreciated on histopathology (H&E high power).

On clinical examination, the temperature was 100.80°F; pulse was 88 beats per minute; respiratory rate was 22 per minute; blood pressure was 110/70 mmHg; and oxygen saturation level was 96%. The systemic evaluation was normal. Upon local examination, palpation revealed multiple right-sided cervical lymph nodes involving the upper and mid jugular, lower and medical supraclavicular, and lymph nodes in the posterior triangle and supraclavicular region. These lymph nodes were firm, hard in consistency, and mobile, with normal overlying skin. They were non-tender to palpation. No palpable lymph nodes were noted bilaterally in the axillary or inguinal regions. The left-sided cervical lymph nodes were not palpable. Systemic examination did not reveal any abnormalities. Bilateral axillary and bilateral inguinal lymph nodes are not palpable. Tests for syphilis, human immunodeficiency virus, toxoplasmosis, cytomegalovirus, Epstein-Barr virus, and tuberculosis were performed on our patient, including venereal disease research laboratory (VDRL), human immunodeficiency virus, hepatitis B virus, and hepatitis C virus (HHH) assessment, and a fever profile. She also underwent a bone marrow examination to exclude lymphoma. These studies were all negative or normal. She was treated with injection cefepime along with the analgesic support with diclofenac sodium and paracetamol. On day 6, excision of the right-sided cervical lymph node (lymph node from the posterior triangle of the neck, and the sample was sent for histological analysis, which identified necrotizing lymphadenitis with traits suggestive of Kikuchi-Fujimoto lymphadenitis shown in Figure 1 below. As a result, Kikuchi-Fujimoto lymphadenitis was determined to be the diagnosis. After starting nonsteroidal anti-inflammatory medicines, the patient's body temperature returned to normal, and the patient's condition was stable at discharge.

## Discussion

Kikuchi and Fujimoto described histiocytic necrotizing lymphadenitis in 1972 [1]. KFD is a rare, benign disease mainly affecting young individuals; it is characterized by histiocytic necrotizing lymphadenitis. While its exact etiology remains uncertain, various factors have been implicated, including viral infections, autoimmune mechanisms, and genetic predisposition [5,6]. Although few instances have been documented on other continents, Asia is the region where this disease is most common. The most widely recognized theory on its etiology is that it has a viral-autoimmune genesis [7]. Apart from non-Hodgkin lymphomas and other lymphoid malignancies, lymphadenopathies linked to connective disorders like systemic lupus erythematosus, rheumatoid arthritis, Still's disease, and bacterial or viral infections like cat scratch disease, infectious mononucleosis, herpes simplex, human immunodeficiency virus, toxoplasmosis, tuberculosis, atypical mycobacterial lymphadenitis, and others should be ruled out in the differential diagnosis of KFD both on clinical and histological bases [8].

In our patient, hemogram and biochemical parameters were within normal limits except for a raised ESR of 80 mm/hour, highlighting the importance of histopathology in confirming the diagnosis. Most of our patients showed cervical lymphadenopathy, which aligns with numerous other findings. According to a thorough analysis, unilateral posterior cervical triangle lymph nodes account for 88.5% of cases of lymph node involvement. Additionally, the patient's elevated ESR and anemia are in line with other earlier instances [9]. There is no set course of treatment because the signs and symptoms normally go away on their own in one to four months without causing any major problems. After the afflicted lymph node is removed, fever typically goes down, indicative of the prospective curative advantage of an excisional biopsy as it can be both diagnostic and remove the source of the inflammation [3].

Differential diagnosis can be aided by lymphadenopathy being shown by imaging modalities like contrast-enhanced computed tomography. This was a limitation in our study as our study is centered on the patient's clinical findings, and histopathological reports did confirmation.

## Conclusions

The best method for diagnosing KFD is lymph node biopsy, which was also the basis of diagnosis in this case. Family members should be consoled, and cases must be comforted that the illness is self-limiting. Studies should continue to discover more about KFD because of its remarkable rarity and diverse clinical presentation. Because histopathological assessment might mimic common conditions, such as tuberculous lymphadenitis and lymphoma, it should be considered in these situations to prevent superfluous investigations and improper therapy. Enhanced cognizance of this disorder among medical professionals is essential to guarantee prompt identification and the best possible results for impacted individuals.

## References

[1] Baenas DF, Diehl FA, Haye Salinas MJ, Riva V, Diller A, Lemos PA (2016). Kikuchi-Fujimoto disease and systemic lupus erythematosus. Int Med Case Rep J.

[2] Ahmed Z, Quadir H, Hakobyan K, Gaddam M, Kannan A, Ojinnaka U, Mostafa JA (2021). Kikuchi-Fujimoto disease: a rare cause of cervical lymphadenopathy. Cureus.

[3] Mahajan VK, Sharma V, Sharma N, Rani R (2023). Kikuchi-Fujimoto disease: a comprehensive review. World J Clin Cases.

[4] Mathew LM, Kapila R, Schwartz RA (2016). Kikuchi-Fujimoto disease: a diagnostic dilemma. Int J Dermatol.

[5] Pai VD, Jadhav RR (2015). Kikuchi's disease: a diagnostic dilemma. Indian J Surg.

[6] Kim HY, Jo HY, Kim SH (2021). Clinical and laboratory characteristics of Kikuchi-Fujimoto disease according to age. Front Pediatr.

[7] Yu HL, Lee SSJ, Tsai HC (2005). Clinical manifestations of Kikuchi’s disease in southern Taiwan. J Microbiol Immunol Infect.

[8] Hutchinson CB, Wang E (2010). Kikuchi-Fujimoto disease. Arch Pathol Lab Med.

[9] Kucukardali Y, Solmazgul E, Kunter E, Oncul O, Yildirim S, Kaplan M (2007). Kikuchi-Fujimoto Disease: analysis of 244 cases. Clin Rheumatol.

